# Hypoxia Inducible Factors as Central Players in the Pathogenesis and Pathophysiology of Cardiovascular Diseases

**DOI:** 10.3389/fcvm.2021.709509

**Published:** 2021-08-10

**Authors:** Emilio Y. Lucero García Rojas, Cleva Villanueva, Richard A. Bond

**Affiliations:** ^1^Department of Pharmacology and Pharmaceutical Sciences, University of Houston, Houston, TX, United States; ^2^Instituto Politecnico Nacional, Escuela Superior de Medicina, Mexico City, Mexico

**Keywords:** hypoxia, cardiovascular, oxygen, hypertension, stroke, sepsis, heart failure, hypoxia inducible factors

## Abstract

Cardiovascular (CV) diseases are the major cause of death in industrialized countries. The main function of the CV system is to deliver nutrients and oxygen to all tissues. During most CV pathologies, oxygen and nutrient delivery is decreased or completely halted. Several mechanisms, including increased oxygen transport and delivery, as well as increased blood flow are triggered to compensate for the hypoxic state. If the compensatory mechanisms fail to sufficiently correct the hypoxia, irreversible damage can occur. Thus, hypoxia plays a central role in the pathogenesis and pathophysiology of CV diseases. Hypoxia inducible factors (HIFs) orchestrate the gene transcription for hundreds of proteins involved in erythropoiesis, glucose transport, angiogenesis, glycolytic metabolism, reactive oxygen species (ROS) handling, cell proliferation and survival, among others. The overall regulation of the expression of HIF-dependent genes depends on the severity, duration, and location of hypoxia. In the present review, common CV diseases were selected to illustrate that HIFs, and proteins derived directly or indirectly from their stabilization and activation, are related to the development and perpetuation of hypoxia in these pathologies. We further classify CV diseases into acute and chronic hypoxic states to better understand the temporal relevance of HIFs in the pathogenesis, disease progression and clinical outcomes of these diseases. We conclude that HIFs and their derived factors are fundamental in the genesis and progression of CV diseases. Understanding these mechanisms will lead to more effective treatment strategies leading to reduced morbidity and mortality.

## Introduction

The industrialization era and the concomitant rise in life expectancy of the past 70 years, has brought a transition to where cardiovascular (CV) diseases are the predominant pathology (1). In the US, the prevalence of CV diseases in adults ≥ 20 years of age is 48% and increases with age (2). CV diseases are also responsible for one third of all deaths, the highest among all disorders. Moreover, Fryar et al. (3) show a staggering 47% of the population with at least one of the three key risks factors (high blood pressure, high cholesterol, or smoking) for developing a CV disease. Although lifestyle changes and current pharmacological treatments have slowly decreased overall mortality for the past 10 years (2), the lack of understanding of the triggering mechanisms that lead to CV disorders keep the incidence, prevalence and mortality at the top of the epidemiologic charts. Recent discoveries in the cellular mechanisms of oxygen sensing, particularly the Hypoxia Inducible Factors (HIFs) are shared by multiple pathologies, including CV diseases. This is a logical association as the main phenomenon in many diseases is a decreased nutrient and oxygen supply under normal or increased demand. Therefore, the mechanisms along the HIF pathway unveil a core pathogenic event in CV diseases that could lead to the development of preventive and therapeutic strategies in CV disease. To provide a full picture to the reader on the relevance of oxygen and its relationship with CV diseases, we will first define important aspects of oxygen physiology, then we will focus on the most relevant pathologic CV states related to a decrease in oxygen supply (hypoxia) and discuss the role of HIFs in the pathophysiology of CV diseases.

## Oxygen as the Central Molecule of Life

The rise of atmospheric oxygen concentrations on earth around 2.1 billion years ago triggered the evolutionary diversification and expansion of aerobic organisms (4). This event contributed to more complex life forms that ultimately developed intricate oxygen delivery networks (respiratory systems) to fulfill the metabolic needs of complex organisms such as the human body. Oxygen, therefore, plays a fundamental role in metabolism, and respiration of CV and all other tissues (5). Under this evolutionary process, we have also developed oxygen sensing mechanisms and adaptation pathways to compensate and survive changes in oxygen concentration (6).

The earth atmospheric gas mixture is composed of 78% nitrogen, 21% oxygen, and 1% argon with traces of carbon dioxide (CO_2_), hydrogen and other gases. At sea level, the atmospheric total gas pressure is 760 mmHg, therefore, the partial pressure of Oxygen (P_O2_), also known as oxygen tension, is 21% of 760 mmHg, or 159 mmHg. Diffusion of oxygen is the net movement from a higher pressure to a lower pressure region. In mammals, a convective system is additionally required for efficient oxygen transport and exchange as the differential pressure between the alveoli and blood is small. Thus, a ventilatory system capable of delivering and removing air from the alveoli, together with a circulatory system that continually supplies deoxygenated blood to the lungs (perfusion), has been developed to comprise the respiratory system and successful oxygen exchange. This transport mechanism favors simple diffusion of oxygen through the gas exchange barrier, the alveoli, with a P_O2_ of 100 mmHg, to the blood. According to Henry's law, the concentration of a gas dissolved in a liquid is proportional to its partial pressure in the gas phase. Therefore, the concentration of oxygen in blood (Ca_O2_) is directly proportional to the alveolar P_O2_ and ranges from 80 to 100 mmHg. The gradient between the alveoli P_O2_ and the arterial partial pressure of oxygen (Pa_O2_) in physiological conditions favors oxygen flow from the alveoli to the blood. Once oxygen is present in the blood, a specialized protein that reversibly binds to oxygen, hemoglobin, can efficiently distribute the gas to all tissues according to their metabolic needs.

Each tissue has a unique metabolic rate that confers its surrounding milieu with its own P_O2_. Hypoxia occurs when the metabolic needs of a tissue are not met because oxygen demand exceeds supply (6). Thus, hypoxic tissue has a low P_O2_ under increased metabolic demand and/or reduced supply. In CV diseases, the Pa_O2_ may not reflect tissue oxygen supply, or tissue oxygenation. A relevant example is shock, where the contractile responses of blood vessels to blood pressure shifts is impaired, thereby decreasing O_2_ delivery to all tissues. Thus, an important goal in shock is the maintenance of blood pressure and monitoring of O_2_ by gasometry. Even with a slight hypoxemia, a septic shock patient can suffer from irreversible hypoxic damage to vital organs. In fact, shock remains as the primary cause of death in intensive care units (7). Recently, emerging evidence suggested measuring mitochondrial oxygen tension as an alternative to gasometry. The rationale for this is mitochondria are the ultimate destination of oxygen utilization (8). Therefore, a precise understanding of the location of the hypoxic insult is relevant to develop a more accurate measurement of regional hypoxia.

## Physiological Regulation of Oxygen Transportation in Blood

The high electronegative nature of O_2_ works as an electron acceptor element that ultimately allows for highly efficient energy generation (in the form of ATP production) from oxidative phosphorylation at the mitochondria of all human cells. For this to happen, O_2_ must first be transported to virtually all tissues of the human body. Since O_2_ solubility in the blood is very low (solubility coefficient = 0.0003), other evolutionary mechanisms such as hemoglobin, were designed to improve transportation and delivery of O_2_ to the tissues.

**Figure 1:**
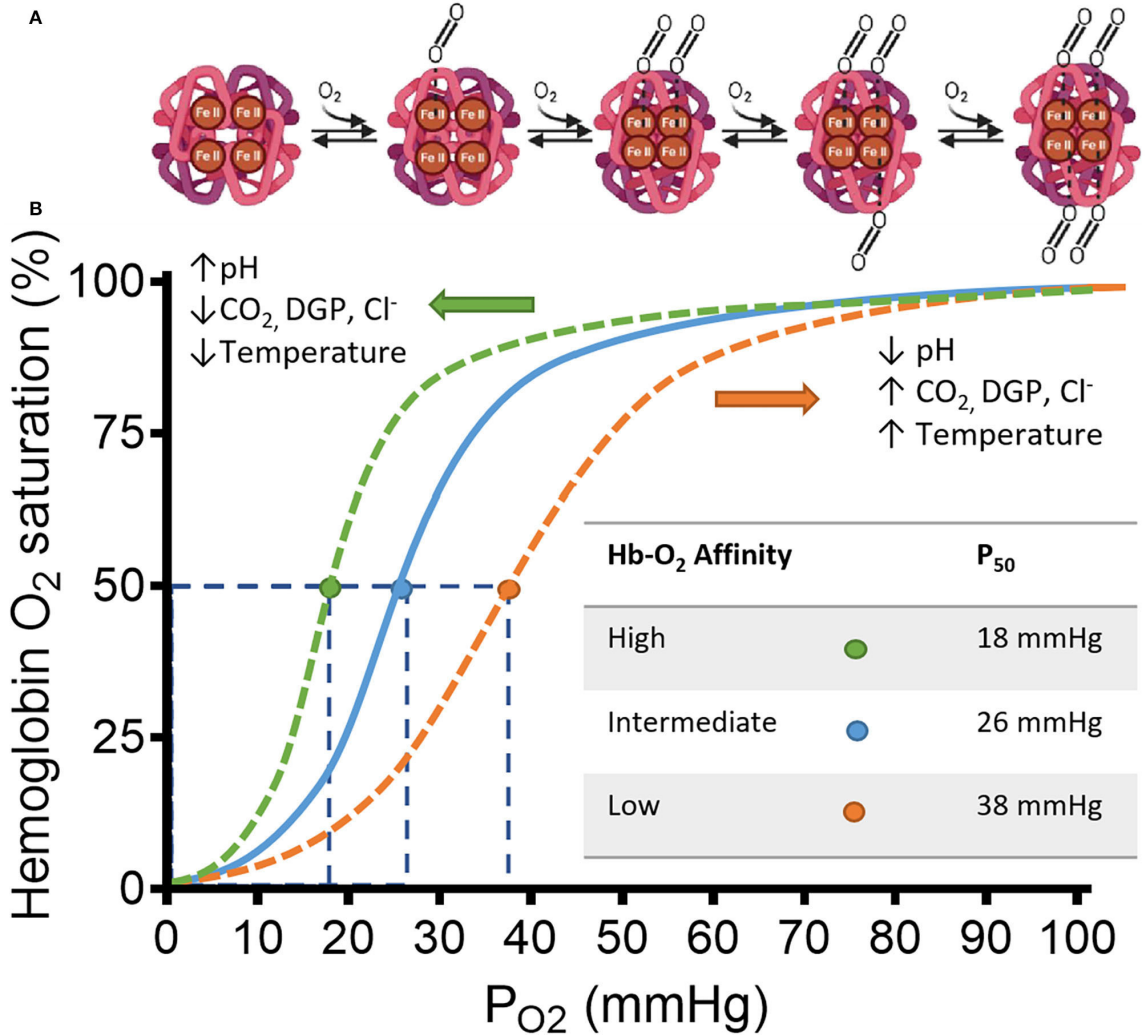
Schematic representation of hemoglobin (Hb) and its affinity for Oxygen (O_2_) in a classic HB-O_2_ dissociation curve. **(A)** The Hb structure has two α- and two β-subunits (light and dark magenta, respectively). Each subunit has a heme residue with Fe^+2^. Transition from deoxy-Hb (“T” state) to oxy-Hb (“R” state) is represented from left to right. Increasing concentrations of O_2_, changes the structure of hemoglobin to “R” state allowing the cooperative binding of O_2_. The transition starts by displacement of H_2_O from the distal region of the binding pocket by a diatomic ligand such as O_2_. Once the O_2_ is sequestered at the distal pocket, the proximity with Fe^+2^ from the heme group allows O_2_ to bind covalently. Finally, minor electrostatic rearrangements stabilize the new conformation. After this process, the hydrophobic interactions between the other subunits are weakened facilitating bonding between O_2_ and the unliganded hemes of the same Hb. **(B)** Hb saturation curve shows changes in affinity for O_2_ by multiple factors. Under low pH, the C-terminal residues of the globin β-chains are protonated, which facilitates O_2_ release from heme by shifting the Hb conformation to the low-affinity T-state (12). Similarly, CO_2_, Cl^−^, and 2,3-diphoglycerate DPG, bind to multiple cationic residues on the N- and C-termini of the α- and β-chains that ultimately stabilizes the T-state conformation of Hb (13, 14). The specific allosteric shift is represented by the orange dashed line displaced to the right from the continuous blue line. The orange dot shows a reduced affinity observed by a higher *P*_50_-value. The opposite is also true, where the allosteric shift to the left by the above-mentioned factors (represented by the green dashed line) increases the affinity of O_2_ for Hb, shown by the green dot and the smaller *P*_50_-value. Adapted from Storz (15).

Hemoglobin (Hb) is a heterotetrameric protein formed by two α- and two β-globin chains of 141 and 146 amino acid residues, respectively, that form two different dimer subunits. Each globin has a heme group characterized by an Iron ion at the center of a porphyrin ring that allows reversible O_2_ binding thus allowing four O_2_ molecules to bind. Hb transitions between a deoxygenated (deoxyHb), low-affinity, tensile “T” state and an oxygenated (oxyHb), high-affinity, relaxed “R” state. A detailed explanation on the structure and binding dynamics of Hb has been reviewed elsewhere (9–11). The binding of O_2_ to a single heme increases the affinity of other O_2_ molecules for the remaining hemes until Hb saturation. The opposite is also true, the release of O_2_ by a single heme forms a saturated Hb and reduces the affinity for the remaining O_2_-bound hemes. This is known as cooperative ligand binding (positive cooperativity) or homotropic allostery and is often represented as the classic sigmoidal Hb-O_2_ dissociation curve plotted as S_O2_ against P_O2_ (Figure 1) (15).

Diatomic ligands such as nitric oxide (NO), an endogenous signaling molecule, and carbon monoxide (CO), a byproduct of cellular metabolism, can competitively displace O_2_ from the heme group. In fact, the overall association equilibrium constants of NO and CO for the heme group are 1 × 10^8^ and 1 × 10^4^ times higher, respectively, than O_2_ (16, 17). The resultant higher affinities in both gases are catastrophic for O_2_ transportation by Hb as the O_2_ concentration available in blood (0.3 mLO_2_/dL) does not meet the metabolic demand of the whole body (≈250 mL/dL in a 70 Kg human). Unlike the heme group alone, the globin structure can help differentiate the unique electrochemical properties of O_2_; O_2_ undergoes an “electrostatic” discrimination by donation of hydrogen bonds increasing its affinity for heme (18).

Other regulatory mechanisms of the Hb-O_2_ affinity are ligands that bind to a distinct region of the globin subunits separate from heme. The first evidence of this affinity modulation was the Bohr effect, where the pH acidity and CO_2_ are inversely related to Hb-O_2_ affinity; thus, a highly acidic pH decreases Hb-O_2_ affinity and vice versa. This type of modulation is known as heterotropic allostery (or classical allosteric modulation) and tightly regulates oxygen release from red blood cells to metabolically active tissue upon changes in the tissue microenvironment (Figure 1).

Mutations on key amino acids involved in O_2_ binding to Hb as well as the expression of other isoforms by globin genes encoding for the α- and/or β subunits also modify the affinity for O_2_ (19, 20) [a detailed list of Hb mutations and clinical consequences in humans can be found elsewhere (21)]. There are 3 α-like globin genes: *HBZ* (ζ-globin), *HBA1*(α1-globin) and *HBA2*(α2-globin); and 5 β-like globin genes: *HBE1* (ε-globin), *HBG1* (Aγ-globin), *HBG2* (Gγ-globin), *HBD* (δ-globin) and *HBB* (β-globin). Diseases that affect the expression of globins, such as sickle cell syndrome and β-thalassemia, or that affect the amount of total Hb, anemia for example, are deficient in O_2_ transportation, and thus induce chronic hypoxic states.

Myoglobin (Mb) is also an O_2_-binding protein expressed mainly in the striated muscle. As a monomeric protein, Mb can only bind one oxygen molecule, although with greater affinity than Hb. This feature favors O_2_ diffusion and storage to the muscle as well as guarantees O_2_ supply for myocytes. Mb, therefore, is vital in hypoxic states, such as exercise, high altitude or water diving, and as a compensatory mechanism by increasing its expression to efficiently store and supply O_2_ to metabolically active tissues (22–24).

In summary, O_2_ transportation in blood requires Hb to meet the demands of all tissues. Under physiological conditions, O_2_ availability is proportional to the metabolic activity of a tissue as CO_2_, lactate, acidic pH, and temperature increase shifting the R-T state equilibrium to favor O_2_ unloading. The continuous O_2_ usage by tissues generates a constant pressure gradient favoring diffusion of O_2_ to the perfused tissue from the capillary blood. Under physiological hypoxic states, however, other mechanisms, such as overall expression, changes in the isoforms or mutations on Hb or/and Mb, as well as vascular tone regulation take place for an optimal handling and delivery of O_2_.

## Oxygen Metabolism

Eukaryotic cells have evolved in an O_2_-rich environment for millions of years and, thus, have efficiently adapted their metabolic needs using the chemical properties of O_2_ to produce energy in the form of ATP (aerobic metabolism). Mitochondria, a double-membraned organelle, produces most of the ATP (90%) using first the tricarboxylic acid (TCA) cycle as the enzymatic machinery in the mitochondrial matrix to metabolize Acetyl CoA [reviewed elsewhere (25)]. Under the TCA cycle, electrons from carbon oxidation are transferred to high-energy bearing molecules (NADH, FADH_2_), that then serve as electron donors for the oxidative phosphorylation (OXPHOS) process. This process relies on the activity of the ATPase synthase (complex V) together with the electron transport chain (ETC) to generate ATP [reviewed in (26, 27)]. Part of the ETC (complexes I, III and IV) pump protons (H^+^) from the matrix to the mitochondrion intermembrane space against their electrochemical gradient using the energy harvested from the electron transfer within complexes by a series of redox reactions. Finally, complex IV (also known as cytochrome c oxidase [Cco]) reduces O_2_ using 4 electrons and 2 H^+^ to form two water molecules. The differential electrochemical gradient drives H^+^ back to the matrix through complex V which uses this gradient to synthesize ATP from ADP and phosphate (Pi^−^) (28, 29). The OXPHOS is, therefore, the main source of energy and depends heavily on intracellular [O_2_]. Decreases in intracellular [O_2_] in hypoxic or anoxic states delay the electron transfer capacity in all complexes. The partially reduced complexes become inefficient at pumping H^+^, decreasing the electrochemical gradient of protons. Ultimately, ATP generation is reduced as less protons are coupled with complex V needed for ATP synthesis.

Most of the oxygen consumption in the mitochondria (~98%) is coupled to the ETC (30). However, some of the electrons transferred through the ETC (~2%) are leaked back into the matrix and react with one of the two unpaired electrons of O_2_ forming superoxide (${\text{O}}_{2}^{•-}$), a central free radical in reactive oxygen species (ROS) formation (31). Free radicals are short-lived intermediates with an unpaired electron in the external orbit making them highly reactive with their environment [extensively reviewed elsewhere (32, 33)]. Up to 90% of ROS production in the cell takes place in the mitochondria (34) as would be expected due to the high amount of overall O_2_ consumption. Other extramitochondrial generators of ROS are Cytochrome P-450 in the endoplasmic reticulum, microsomes and peroxisomes, xanthine oxidase in the cytoplasm, and the NADPH oxidase (NOX/Duox) family members, which under high and normal [O_2_], are thought to transport single electrons across biological membranes to generate ${\text{O}}_{2}^{•-}$ (35, 36). The ROS have proven relevant in the regulation of multiple cellular functions such as oxidative stress regulation, enhancement of signal transduction, sensing of intracellular O_2_ tension, among others (37). Increased supply of O_2_ (hyperoxia) rises ROS production as more electrons leak from the ETC and partially reduce O_2_ (38). Seemingly paradoxical, hypoxia can also augment ROS level by impairment of the ETC which also enhances electron leakage (32, 39–41). This paradoxical phenomenon appears to be tissue specific. The clinical relevance of such changes in oxygenation are manifest in multiple pathologic states where long term exposure to free radicals can induce “oxidative damage” in DNA and mutagenesis as the first step in carcinogenesis. Intracellular O_2_ levels, therefore, are inherently associated to ROS production and maintaining optimal cellular function. This tight association makes ROS suitable candidates for sensing of intracellular [O_2_] as will be discussed in the next section.

## Oxygen Sensing

The function of a sensor, strictly speaking, is to detect a change in the system that ultimately generates a response (signal) to maintain the biological homeostasis of the system. It is reasonable, then, to define first the O_2_ range at which cells optimally operate as normoxia (homeostasis). It is widely reported that cellular normoxia ranges between 10 and 2% (76–15.2 mmHg), hypoxia would be between 2 and 0.5% of O_2_ tension (15.2–3.8 mmHg) and anoxia would be below 0.5% (<3.8 mmHg). However, this is a generalization as each tissue possesses a different threshold for hypoxia at which the molecular machinery is activated to compensate for the bioenergetics of the cell. For example, the carotid body, a peripheral chemoreceptor that signals to the medulla to regulate ventilation, senses hypoxia when the O_2_ tension falls below 8% (<60 mmHg) by the chemosensitive glomus cells in mammals (42, 43). Conversely, O_2_ tension in bone tissue under physiological conditions (physioxia) ranges from 0.6 to 3% (~4.5–23 mmHg) depending on the regional vasculature in the bone (44). Decreased bone oxygenation by disruption of blood delivery, as happens in fractures, activates bone mass repair and growth (45). An extensive and well-documented review on the oxygen distribution across all organs can be found in (6). Moreover, acute, chronic, or intermittent changes in tissue oxygenation modify the cells' activity (46). Thus, the threshold of each tissue to hypoxia is finely tuned by the tissue vascularization, metabolism, function, and time of exposure to low [O_2_]. Such complex regulation of cells in hypoxic states has made it difficult to determine all the sensing mechanisms involved in the threshold for the hypoxic response of each tissue. Nevertheless HIFs, key regulators of the adaptive responses to cellular hypoxia in physiologic and pathologic conditions in all tissues, have improved our understanding of the oxygen sensing mechanisms (47–50).

**Figure 2:**
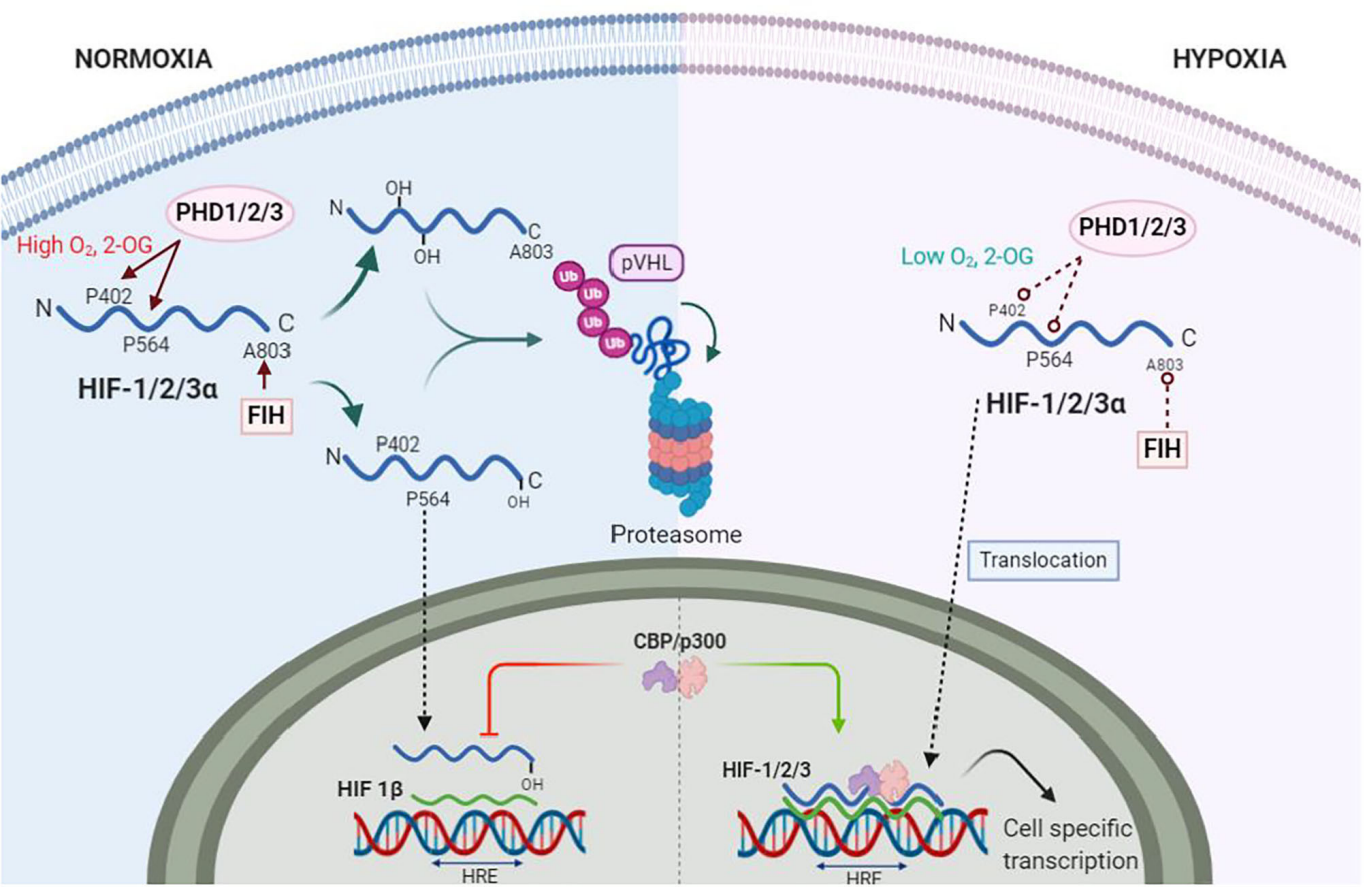
Hypoxia Inducible Factors (HIFs) under normoxia and hypoxia. During normoxia proline residues 402 and 564 (P402, P564) of HIF-α (blue wave) are hydroxylated by prolyl-hydroxylase 1 and 2 (PHD1, PHD2), whereas PHD3 hydroxylates only P564. The factor inhibitor of HIF (FIH) hydroxylates asparagine residue A803, which inhibits binding of nuclear protein CREB (CREB) and p300 s. PHDs and FIH are dependent on oxygen (O_2_) and 2-Oxoglutarate (2-OG) concentrations. Hydroxylated HIF-1α is ubiquitinated by von Hippel Landau (VHL) ubiquitin ligase complex for proteasomal degradation. Under hypoxia, PHDs and FIH become inactive, allowing HIF-α stabilization and translocation to the nuclei. Here, HIF-α dimerizes with HIF-1β (green wave). The dimer is then termed HIF-1/2/3 and binds to the hypoxia response elements (HRE) of specific genes in the DNA to transcribe thousands of genes.

First discovered almost 3 decades ago, HIFs are transcription factors expressed as heterodimers formed by one of three α isoforms (HIF1-3α), with HIF-1α being the most universally expressed (51), and a β subunit [also known as aryl hydrocarbon receptor nuclear translocator (ARNT)] constitutively expressed in all tissues (52). During normoxia, the HIFα subunits undergo post-translational hydroxylation by the dioxygenase family of enzymes to regulate the activity and accumulation of HIFα. The activity of HIFα is dampened, on one hand, by the hydroxylation of an asparagine residue in its C-terminal by the dioxygenase Factor Inhibiting HIF (FIH). This specific hydroxylation blocks the interaction with the nuclear coactivators p300/Creb binding protein (CBP) (53). The production of HIFα, on the other hand, is regulated by the hydroxylation of specific proline residues by prolyl hydroxylases (PHDs). The hydroxylated prolines enable the interaction with the von Hippel-Lindau tumor suppressor (pVHL), an E3 ubiquitin ligase, that ultimately recruits the HIFα subunits to the proteasome for degradation (Figure 2) (54–56). Out of the 3 isoforms of PHDs (PHD1-3), PHD2 is the most abundant and relevant in hypoxia (57, 58).

The catalytic activity of both dioxygenases FIH and PHDs are dependent on the intracellular [O_2_]. Dioxygenases also use 2-oxoglutarate (2-OG), a product of the TCA cycle metabolism, and Iron, a sensitive redox element highly reactive to O_2_, as cofactors to hydroxylate HIFα (59). Together with the intracellular [O_2_], the reduced Iron (Fe^2+^) is required at the catalytic site for the oxygen sensing activity of dioxygenases (60). The reducing agent ascorbate, another product of the TCA cycle, is responsible for the constant reduction of Iron, thus, keeping dioxygenases active. In hypoxia, however, lower ascorbate production by the TCA cycle modifies Iron redox equilibrium to an oxidized state (Fe^3+^) that, ultimately, decrease Fe^2+^ bioavailability to activate PHD and FIH. Moreover, ascorbate and 2-OG as intermediates of the TCA cycle could be the link between the aerobic metabolic activity and HIF activity in hypoxia as their production is decreased under hypoxic states (61, 62). The previously described biochemical process is thought to be the mechanism by which oxygen is sensed by dioxygenases in cells (63). Thus, dioxygenases are the true O_2_ sensors whereas HIFs are the activation signal that induces an effect.

Other O_2_ sensing mechanisms have been proposed to regulate cellular function (64). Of particular interest are the mitochondrial-produced ROS as determinants of HIF activity. Some data suggest that the increase in ROS during hypoxia stabilizes HIF-1α and−2α (65). Moreover, elevated ROS increases HIF-1 levels that favor tumorigenesis under hypoxia (66). Finally, there is evidence that PHD inhibition and subsequent HIF-1α stabilization is mediated by ROS increments during normoxia and hypoxia (67). These data, altogether, suggest ROS as part of the O_2_ sensing mechanisms of the cells. However, conflicting evidence do not favor this hypothesis [reviewed by Movafagh et al. (68)]. Whatever the case might be, is clear that O_2_ availability influences both systems, ROS formation and HIF activity. Thus, is reasonable to infer that multiple sensors have been developed by the cells for a tight regulation of intracellular [O_2_] and alterations of these sensor systems in hypoxic conditions can lead to disease.

Inactivation of dioxygenases by hypoxia allows HIFα subunits to stabilize, translocate and accumulate in the nuclei to dimerize with the HIFβ subunit (69). The HIF dimer recognizes and binds to the targeted DNA sequences within the Hypoxia Response Element (HRE) and recruits p300/CBP coactivators to begin transcription (Figure 2). More than a thousand genes involved in a myriad of functions such as erythropoiesis, glucose transport, angiogenesis, glycolytic metabolism, ROS handling, cell proliferation and survival among others, are directly transactivated by HIFs, particularly HIF-1 (70, 71). Such versatility of HIFs to activate genes for multiple tasks underline the importance of HIFs as a regulator in hypoxia. Moreover, other insults commonly observed in CV diseases, such as inflammation, can further activate HIFs and downstream genes. We will now discuss the relevance of HIFs and its most relevant target genes related to specific CV related pathologies. For this, we have classified CV diseases based on the duration of the hypoxic insult (acute vs. chronic) to better understand the timely differences of HIFs regulation.

## Acute Pathologic Hypoxic States

**Figure 3:**
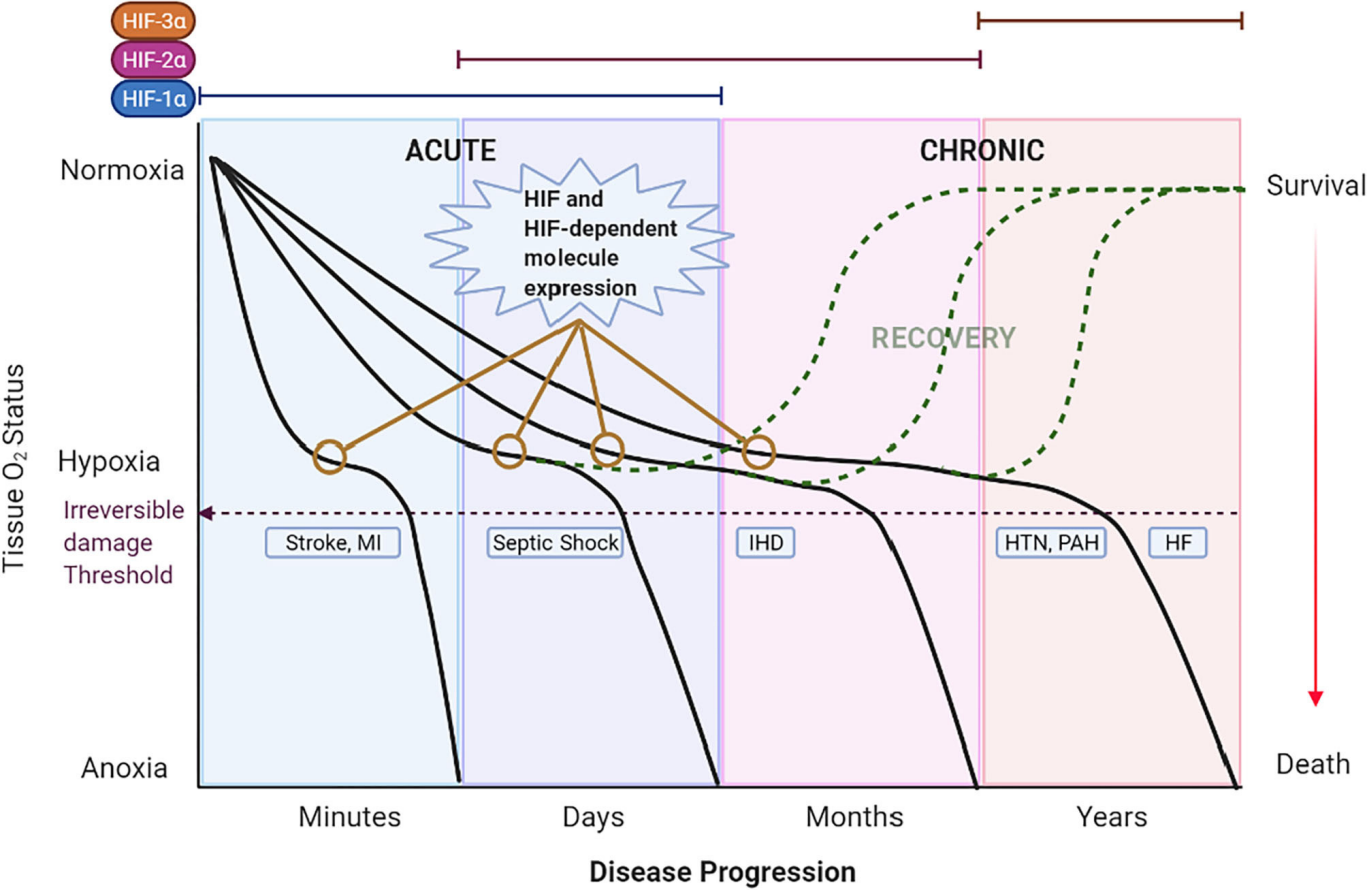
Theoretical schematic representation of the temporal expression of HIFs and the impact of hypoxia in CV diseases across time. The left 2 panels show acute hypoxic states [i.e., Stroke, myocardial infarction (MI) and Septic Shock] lasting minutes to days. A rapid decrease in O_2_ (first panel) induces severe hypoxia/anoxia and irreversible damage in minutes to hours leading to death or permanent disability (purple dashed line). The second panel represents septic shock as the hypoxic state that lasts days. Under these acute hypoxic states, HIF-1α is stabilized, translocated to the nucleus and bound to HIF-1β to form a dimer termed HIF-1. HIF-1 activates the transcription of multiple genes as an adaptation response. Eventually, HIF-1α expression is switched to other HIFs; HIF-2α and HIF-3α in chronic hypoxic states. On the right 2 panels the chronic hypoxic states [i.e., Hypertension (HTN), Pulmonary Arterial Hypertension (PAH) and Heart Failure (HF)] are represented. Here, the sustained mild hypoxia induces long-term adaptive mechanisms triggered by HIFs that, ultimately, become detrimental and lead to death. Expression of HIF and HIF-dependent genes (represented by yellow lines with empty circles) are observed in the early stages of CV diseases. Detection of such genes at an opportune time might allow for the early treatment and recovery of the patient (dashed green curves).

Acute pathologic hypoxic states are produced by a sudden reduction of blood flow to a tissue that can last minutes to days. Several mechanisms, including increased oxygen transport and delivery as well as increased blood flow, are triggered to compensate for the hypoxic state. If hypoxia prevails over the compensatory mechanisms, irreversible damage is generated (Figure 3). The compensatory, and later maladaptive, cellular mechanisms involving HIFs and its targeted genes in these acute hypoxic pathologies suggests the importance of such molecules in the origin and evolution of these CV diseases. Moreover, the role of HIFs in acute hypoxic states reveals possible mechanisms that can be targeted to avoid irreversible damage. This section focuses on the involvement of HIF under acute hypoxia present in the selected CV pathologies of stroke, sepsis/septic shock, and coronary artery disease/ischemic heart disease. Understanding HIFs relationship with CV pathophysiology could lead to the development of new therapeutic strategies to reduce morbidity and mortality.

### Stroke

Stroke is the second cause of death worldwide. Ischemic stroke is the most prevalent type representing around 85% of cases (72). One out of five stroke patients have a recurrent stroke within the next 5 years and half of the survivors remain disabled (73). Moreover, the cause of stroke is unknown in around 30% of patients (72, 73), which directly affects the diagnostic capacity to avert increased morbidity and mortality. Thrombolysis continues to be the treatment of choice for ischemic stroke. However, the window-time of treatment is up to 4.5 h after the onset of ischemic stroke. If thrombolysis is delayed, ischemic stroke transforms into hemorrhagic stroke, with mortality reaching 80% (74).

The trigger of the ischemic stroke cascade is tissue hypoxia, caused by the occlusion of a cerebral artery, and shows multiple damage degrees in part based on the tissue ATP levels. For example, the ischemic core has an ATP reduction of around 85%, whereas the area around ischemic core, the penumbra, ATP drops 30–50%. Penumbra tissue, although affected, is potentially salvageable (73, 75) if hypoxia is reversed in time. Estimations on necrosis volume across time show an increase of 20% in the necrotic area within 3 h of blood flow interruption (76).

The role of HIFs in the pathophysiology of cerebral ischemia is known since the 90s (77). However, there is controversy to whether HIF induction has negative or positive effects on stroke. For instance, neuronal HIF-1α knockout mice show neuroprotection in a model of stroke (78). Similarly, HIF-1α/-2α double knockout mice under medial cerebral artery ischemia/reperfusion show decreased infarct volume, cerebral edema, and expression of anti-survival genes (i.e., *BNIP3, BNIP3L*, and *PMAIP1*), 24 h after reperfusion. However, no protective effects are shown in these mice when reperfusion period is extended to 72 h (79). This suggests that early activation of HIFs have deleterious effects on cerebral ischemia, whereas late activation could be beneficial. Accordingly, rats submitted to 2 h of middle cerebral artery and carotid occlusion were transplanted with bone mesenchymal stem cells (BMSC) overexpressing HIF-1α at 24 h after reperfusion. This approach reduced the infarct volume and improved neurological function (80). Other studies, using a limb remote ischemic preconditioning model in rats [previously proven to increase HIF-1α systemically (81)] as a prior stimulus to modulate brain HIF-1α/-2α expression during stroke, observed a decreased inflammatory response and brain injury (82). Similarly, using PHD inhibitors for HIF-1α stabilization 6 h prior to stroke was neuroprotective (83). These studies demonstrate the relevance of HIFs, particularly HIF-1α, as the key regulators in the evolution of stroke across time. Consistently, stroke patients recently admitted to the hospital showed a high expression of HIF-1α. This expression was strongly positively correlated with the National Institute of Health Stroke Scale (widely used scale to quantify stroke severity based on a clinical assessment) (84). Thus, it seems that the detrimental physiological effects observed by the increased expression of HIF-1α during the early stage of stroke is similar in rodents and humans.

#### Regulation of HIF-1α in Stroke by miRNA

Several microRNAs (miRNAs), known for their post-transcriptional regulation of gene expression, have been associated with stroke, influencing neural cell survival, and regulating brain inflammation though the stimulation or inhibition of HIFs (85). Ischemic preconditioning (IPC) consists in submitting a subject to short hypoxic episodes before a severe ischemic event, in order to reduce the negative effects of subsequent hypoxia. The family of miRNAs-200s increase in the cerebral cortex of mice submitted to IPC before cerebral ischemia (86). This family of miRNA inhibit PHD expression which increases the stability and activity of HIF-1α. Particularly, stroke patients showed better outcomes when higher miRNA-210 levels were detected in blood within 7 days after stroke (87). Thus, the high miRNA-210 levels produce a type of preconditioning event that allows for neuroprotection in stroke patients.

### Sepsis/Septic Shock

According to the Third International Consensus Task Force (Sepsis-3) sepsis is defined as a life-threating organ dysfunction caused by a dysregulated host response to infection, whereas septic shock is a subset of sepsis in which profound circulatory, cellular and metabolic abnormalities are associated with greater risk of mortality than sepsis (88). Septic shock remains the leading cause of death in critically ill patients with 30% of patients dying within the first 3 days (7). A recent meta-analysis further showed that the average mortality of septic-shock was 34.7 and 38.5% at 30 and 90 days, respectively. This mortality rate has been almost the same in the last 10 years (89). Thus, it is critical to better understand the molecular mechanisms involved in this pathology to improve our detection methods of regional hypoxia and develop greater prognostic value.

The pathophysiologic features (i.e., immune response, hemodynamic, and metabolic states) that define septic shock across time are paradoxically opposite during the early vs. the late phase. The early phase is characterized by a hyperdynamic vascular response, together with high pro-inflammatory cytokine release and a shift from oxidative to aerobic glycolytic metabolism (Warburg effect). Conversely, the late phase is defined by a hypodynamic vascular response, anti-inflammatory cytokine release and a hypometabolic state (90). Recent evidence on the metabolism of multiple immune cells, the main mediators of inflammation in sepsis and septic shock, shows the Warburg effect at the crossroads between inflammation and metabolism (91, 92). First discovered in cancerous cells (93), the Warburg effect refers to the switch of energy production from oxidative phosphorylation in the mitochondria to glycolysis with rapid formation of lactate even in non-hypoxic conditions (94). In multiple immune cells, including T cells, neutrophils, dendritic cells, natural killer cells and macrophages (95–98), the Warburg effect is observed after activation of toll like receptor 4 (TLR4) by lipopolysaccharide (LPS), the main antigen that induces sepsis (99, 100). This ensures faster ATP generation at the expense of less efficiency to provide for intermediates for cell growth and proliferation (101) that, ultimately, allow for a fast and strong inflammatory response by the immune system to pathogens. Moreover, multiple studies show HIF-1α as an important mediator of the Warburg effect. HIF-1α is activated by LPS in normoxic conditions (102), regulates cytokine expression (103, 104) and leads to a metabolic reprogramming of immune cells (105). Under the metabolic switch during infection, accumulation of the TCA cycle intermediate, succinate, is transported to the cytoplasm and stabilizes HIF-1α (106, 107). Thus, the metabolic changes in the immune system during sepsis as well as the inflammatory response in septic shock are tightly regulated by HIF-1α. Accordingly, deletion of HIF-1α in the myeloid lineage protects mice against LPS-induced mortality blocking the inflammatory response, hypotension and hypothermia (102, 108). Similarly, HIF-1α deletion in T cells increase survival in a rodent model of sepsis (109). Finally, increased HIF-1α activity by PHD3 deletion decreased survival by an overwhelming innate immune response in a rodent model of abdominal sepsis (110). Altogether, this evidence suggests that increased HIF-1α levels in the early phase of sepsis/septic shock is detrimental for survival.

In humans, Textoris et al. (111) observed an increase in HIF-1α mRNA from whole blood samples of patients ongoing septic shock for the first 3 h after diagnosis. Conversely, HIF-1α mRNA levels in blood and protein expression in leukocytes were decreased in patients with severe sepsis (112). This study further showed that the low HIF-1α expression was dependent on the exposure time to LPS in human monocytes: high HIF-1α expression in the acute administration (likely resembling the early phase of sepsis) and low HIF-1α expression under prolonged stimulation. Furthermore, cytokine gene expression was maintained even after the HIF-1α mRNA expression was reduced. Therefore, the increased expression of HIF-1α might initially favor the inflammatory response observed in patients with septic shock which, in the long term becomes maladaptive by mediating a prolonged inflammatory response and increasing the severity of the disease.

### Coronary Artery Disease/Ischemic Heart Disease

The coronary arteries are the main suppliers of blood and O_2_ to the myocardial tissue. Atheroma formation on the endothelial layer of the coronary walls reduce perfusion to the heart [i.e., coronary artery disease (CAD)]. Increased cardiac performance when coronary atherosclerotic lesions are present can induce ischemia in the cardiac tissue leading to decreased blood flow and O_2_ delivery and accumulation of toxic metabolites. This disorder is known as ischemic heart disease (IHD). The characteristic symptom of chest pain induced by the ischemic heart is referred to as angina pectoris and episodes are commonly produced by intense exercise and emotional stressors. Rupture of the atheroma plaque can produce a thrombus that causes intermittent coronary occlusion leading to episodes of unstable angina at rest. Alternatively, severe stenosis from an atherosclerotic plaque or thrombus that obliterates the lumen of the vessel can result in a myocardial infarction (MI). In IHD, the formation of atherosclerotic plaques usually takes decades and, the slow progression of the pathology is reflected as a higher prevalence with age (113). During the plaque development, several episodes of silent ischemia can occur in at least in 50% of the affected population where the cardiac hypoxic tissue activates the transcription of angiogenic genes to stimulate collateral vessel formation (114). This is a compensatory mechanism mediated by HIFs to keep optimal O_2_ delivery to the myocardial tissue. Collateral vessel formation limits the size of infarction and increases survival of patients with MI (115, 116). Therefore, identification of the mechanisms related to the progression of the disease, such as HIFs and downstream targeted genes, can be useful for the development of new therapeutic targets that reduce adverse clinical outcomes and increase survival.

A closer look into HIFs on the adverse outcomes of IHD shows HIF-1α activity as essential for cardioprotection. This has been explored using ischemia preconditioning (IPC) (described in section Stroke) before inducing ischemia reperfusion injury (IRI), a severe cardiac ischemic event observed in infarcted patients that undergo thrombolysis, percutaneous coronary intervention or coronary bypass grafting (117). In rodents, IPC or ambient intermittent hypoxia (as a robust method to induce cardiac hypoxia preconditioning) improved functional recovery and limited infarct size and apoptosis in hearts submitted to IRI. These myocardial protective effects were mediated by HIF-1α as HIF-1α deletion abolished such responses (118–120). Other studies have corroborated this finding by directly overexpressing HIF-1α (121) or increasing HIF-1α stability by cardiac deletion (122–126) or inhibition of PHDs (127–129). These approaches consistently protected the heart against a severe ischemic insult (i.e., MI) shown as decreased infarct size. Additionally, other studies using remote IPC also showed HIF-1α as cardioprotective when a prolonged cardiac ischemic event is induced (130, 131). This protective hypoxic preconditioning is also directly related to the cardiac proliferation of a small subset of cardiac cells. This subset of cells, referred to as hypoxic cardiomyocytes due to their hypoxic surrounding milieu, show a constitutive stabilization of HIF-1α, downregulation of PHDs, decreased ROS production as well as upregulated cyclin genes that, under further hypoxic preconditioning, lead to mitosis and, hence, cardiomyocyte proliferation (132, 133). A strikingly similar cell phenotype with regenerative characteristics has also been reported in humans under an alternative name, cardiac stem/progenitor cells (CSCs) (134). Multiple reports show that CSCs differentiate into cardiomyocytes, vascular or endothelial cells. After cardiac injury, these cells reduce the infarction size by decreasing cardiomyocyte apoptosis and oxidative DNA damage (a marker for cardiac senescence) which favor cell cycle re-entry, and/or by increasing angiogenesis (134, 135). Besides the multiple cardioprotective mechanisms induced by HIF-1α activation during heart ischemia [reviewed by Semenza (136)], an increased capillary density also appears to be highly consistent (121, 127–129). Interestingly, part of the cardioprotective events elicited by the cardiac endothelial HIF-1α are mediated by at least 2 mechanisms: Adenosine production and release (137) and Angiopoietin (124) signaling. All this evidence together shows HIF-1α as an acute regulator, beneficial for the survival response of the heart during harmful ischemic insults.

Analysis of genetic polymorphisms in the human HIF1A locus has shown that CAD patients with a single-nucleotide polymorphism (P582S) showed increased collateral vessel formation (138). Moreover, an initial clinical assessment of patients with CAD showed stable angina as more prevalent in subjects with multiple single nucleotide polymorphisms in the HIF1A locus compared to patients that suffered an MI (139, 140). The stable angina group was also associated with increased coronary collaterals (140, 141). This data shows that polymorphisms activating HIF-1α in humans also mediate cardioprotection. It is important to note that HIF-1α expression is decreased with age (142, 143) and, hence, the cardiac protective effect of patients with CAD undergoing IPC (144) is lost in the aged hearts (145).

There is also plenty of evidence linking adenosine with HIF-1α and its relevance in the pathophysiology of CAD as we will describe next.

#### Adenosine

This purine nucleoside composed by adenine attached to a ribose is critical for the regulation of coronary circulation (146). Adenosine is the metabolic product of ATP dephosphorylation by two enzymes highly expressed in the endothelial vasculature: CD39, also known as ectonucleoside triphosphate diphosphohydrolase, and CD73, also known as ecto 5′-nucleotidase. In a process known as auto-regulation (147), during an ischemic event, these enzymes are overexpressed and favor the rapid metabolism of ATP to produce adenosine (148, 149). The vascular activity of adenosine is then mediated by adenosine receptors (AR). Four AR have been characterized: A_1_, A_2A_, A_2B_, and A_3_ (150). Interestingly, A_2B_, as well as CD73 and CD39, gene transcription is targeted by HIF-1α, thus, establishing the adenosine system essential for survival in ischemic events. Like CD39 and CD73, A_2B_ is predominantly expressed in coronary endothelial cells and activates vasodilatory mechanisms after adenosine binding (151). Mice lacking endothelial HIF-1α lose the cardioprotective effect induced by IPC. This feature is rescued by the administration of adenosine (137). Other studies have also observed loss of cardio-protection by deleting any of the HIF-1α-dependent components related to the adenosine system: CD39, CD73 or A_2B_ (120, 148, 149). This data suggests that the enzymatic machinery to produce adenosine as well as the interaction with the A_2B_ is necessary to increase O_2_ delivery to the heart by increasing blood flow during hypoxia. Increased expression of these components appears to be a compensatory mechanism for cardio-protection during ischemic events. In humans, adenosine levels have been inversely correlated with the presence of obstructive CAD and advanced age (152), perhaps by the age-dependent decrease of HIF-1α. Finally, other small study showed a decrease in adenosine levels after percutaneous transluminal coronary angioplasty in patients with CAD (153). Altogether, the evidence shows the relevance of adenosine production and vasodilatory function in coronary arteries in patients with CAD.

## Chronic Hypoxic States

Under this subset of pathologic conditions there is a reduced oxygen delivery to tissues that can last days to years. We have chosen to discuss systemic and pulmonary hypertension, as well as heart failure as examples of diseases characterized by chronic hypoxic states. The long-term exposure to hypoxia induces a series of genetic adaptations mediated by HIF activation, that can potentially lead to adverse pathologic conditions even in healthy humans living at high altitude. Indeed, sojourners (acclimated lowlanders) and native residents of cities with altitudes >2,500 m above sea level, show increased blood viscosity and high pulmonary vascular resistance as the adaptive response to chronic hypoxemia (154). These adaptive responses are related to increased expression of erythropoietin (*EPO*) and Cu-uptake transporter1 (*CTR1*) genes, respectively. The chronic upregulation of such genes can, then, favor the development of pulmonary hypertension (155–157). Other maladaptive conditions are seen in newborns where birthweight is inversely related to the altitude (the higher the altitude, lower the birthweight), and the higher prevalence of maternal hypertension and preeclampsia in highlanders (158–160). These maladaptive mechanisms have been partially circumvented by gene polymorphisms unique to populations (i.e., Tibetans and Ethiopians) exposed to chronic hypoxic conditions for tens of thousands of years. At least 2 genes, *EPAS1* (codes for HIF-2α) and *ARNT2* (codes for HIF-1β), in the Amhara ethnic group in Ethiopia, and the *EPAS1* and *EGLN1* (codes for PHD2) genes in Tibetans, have polymorphisms responsible for such true adaptive mechanisms. This is translated as normal levels of hemoglobin despite the hypoxic conditions at high altitude (154, 161). Overall, the current evidence shows that chronic hypoxia is detrimental for most of the population and can lead to CV pathologies mediated by HIF-dependent gene expression. Moreover, each tissue is under a different gene regulation scheme by HIFs, creating specific patterns of expression depending on the affected hypoxic tissue (Figure 3). Therefore, in this section we focus on the CV pathologies most affected by chronic hypoxia: systemic hypertension, pulmonary hypertension and heart failure, and their HIF-dependent genetic expression as a mechanism underlying the pathophysiology of these diseases.

### Systemic Hypertension

Known as the silent killer, systemic hypertension (HTN) is currently defined as a systolic blood pressure (SBP) ≥ 130 mmHg and/or a diastolic blood pressure (DBP) ≥ 80 mmHg. The overall prevalence among the US adults aged ≥ 20 is 45.4% (162). Several epidemiologic studies have pinpointed sleep apnea, either obstructive or central, as one of the causes for systemic HTN (163, 164). Certainly, the severity of the apnea has been correlated with the development of HTN in the subsequent 4 years after diagnosis (165). Lack of ventilation (apnea) for up to 45 s comes with oxygen desaturation (SO_2_ ≈ 80%). These single events are repeated dozens to hundreds of times per night leading to chronic intermittent hypoxia (CIH) (166). The resultant hypoxemia is detected by glomus cells, the primary chemosensitive transducers in the carotid body, as small drops in oxygen tension (Pa_O2_ ≤ 70 mmHg) that promote a HIF-mediated response (167, 168). Repetitive stimuli by CIH increases the sensitivity of the glomus cells to detect low O_2_ tension as well as enhances the coupling mechanism between hypoxia and the elicited response in a phenomenon called sensory long-term facilitation (LTF). In turn, the cardiorespiratory center in the brainstem, through the nucleus tractus solitarius and the rostral ventrolateral medulla, increase the vascular tone by boosting the sympathetic activity (169, 170). The sympathetic hyperactivity, translated as increased catecholamine release from the sympathetic system, is a central factor in the development of high blood pressure (171). Together with the CIH, the sympathetic hyperactivity affects all the cellular components of the circulatory system amplifying the severity and complexity of the disease leading to vascular disfunction and low-grade proinflammatory responses. Such complex scenarios have challenged our ability to identify the primary nature of the disease and delay an accurate and timely treatment for hypertensive patients. Yet, in light of the recently characterized HIF pathway and its central relationship with HTN, we speculate that molecules involved in such pathways are deeply related to the pathogenesis of HTN and potentially useful for the early detection of the CV risks in HTN.

#### The HIF Pathway in the Pathophysiology of HTN

Expression of HIF-1α in the carotid body is reduced during normoxia and gradually increases as the Pa_O2_ decreases. Conversely, HIF-2α is highly expressed in the same tissue under normoxia and decreases under CIH (172). Moreover, HIF-1α heterozygous-null mice (hif1a^+/−^) do not develop HTN when exposed to CIH (173), whereas hif2a^+/−^ mice show hypertension and high norepinephrine plasma levels in normoxic conditions (174). This implies that HIF-2α and/or downstream targeted genes are needed for blood pressure regulation to physiologic levels. Conversely, HIF-1α and/or specific downstream targeted genes induce the development and perpetuation of hypertension. Thus, the overall differential regulation of both HIFα subunits in CIH could be interpreted as a ratio (HIF-1α/-2α) where the higher the value, higher the severity of the disease and, thus, increased the risk for CV events.

Prolyl hydroxylase 3 (PHD3) is also involved in blood pressure regulation. As previously mentioned, the increased levels of HIF-1α during hypoxia induce PHD3 expression (175). High PHD3 expression has been observed in the cardiovascular tissue in a HIF-dependent way under hypoxia (176). Since PHD3 activity contributes more to the regulation of HIF-2α than to the regulation of HIF-1α (177), a possible switch mechanism mediated by PHD3 during hypoxia might play a main role in blood pressure regulation. The relevance of PHD3 in blood pressure regulation was demonstrated in mice lacking PHD3 (PHD3^−/−^) that showed enlarged sympathetic ganglia partially mediated by HIF-2α expression. Moreover, PHD3^−/−^ mice had decreased innervation to targeted tissues, with dysfunctional sympathetic responses and hypotension (178). Thus, PHD3 is a fundamental enzyme for the normal development and function of the sympathoadrenal tissue. Subtle changes in either the activity or expression of PHD3, or HIF-2α expression, modify the sympathetic regulation and catecholamine secretion, central to the HTN pathology.

### Pulmonary Arterial Hypertension

Contrary to the dilatory response in the systemic circulation under acute hypoxia, pulmonary arteries constrict as a mechanism to optimize flow to the most ventilated areas of the lungs (179). Prolonged periods of hypoxia, as in high altitude or chronic lung diseases, results in pulmonary vascular remodeling and increased arterial wall stiffness. The structural modifications of the vasculature in such conditions are pulmonary artery smooth muscle cell (PASMC) remodeling, including hyperplasia and hypertrophy, as well as unregulated proliferation of endothelial cells causing plexiform lesions that occlude the vessel lumen. Other changes such as increased vascular tone and decreased reactivity to vasodilators are also present. These progressive changes increase the pulmonary vascular resistance (PVR) and mean pulmonary arterial pressure (mPAP) until pulmonary arterial hypertension (PAH) is developed (i.e., mPAP ≥ 25 mmHg) (180). The development of these pathologic events is clinically translated into dyspnea, chest pain and syncope; very unspecific signs and symptoms that makes the diagnose difficult in early stages. Late detection of PAH increases the risk of mortality with decompensated right heart failure as the most common cause of death (181, 182). Thus, it is important to understand the pathogenic mechanisms which can then be used for early detection of PAH. Next, we discuss recent findings on the crucial influence of HIFs, its modulators, and downstream molecules in the early development of the disease.

#### HIFs in PAH Pathogenesis

Mounting evidence is supporting HIF-1α and HIF-2α as independent factors that contribute synergistically to PAH development. For instance, *in vitro* studies showed HIF-1α highly expressed in human PASMC only under hypoxia, whereas hypoxia-dependent HIF-2α overexpression was only observed in pulmonary artery endothelial cells (PAEC) (183–186). The same pattern was detected in biopsies from patients with PAH compared to healthy controls (184, 187, 188). Studies in other mammals (i.e., rodents) are also consistent with these findings. hif1a^+/−^ mice are protected from left ventricular hypertrophy and right ventricular pressure elevation under hypoxia (189). Importantly, hif2a^+/−^ mice were devoid of the pulmonary hypertensive response to hypoxia (190). Altogether, the evidence suggest that the high expression of both HIF isoforms indicate disease severity. This contrasts with systemic hypertension where CIH induces overexpression of HIF-1α while inhibiting HIF-2α expression, making the elevation of both HIFs specific to PAH. In an effort to categorize the events downstream of HIF activation and their relevance in PAH we will divide them as occurring in the smooth muscle cells (SMC) and/or occurring in the endothelial cells.

##### Pulmonary Artery Smooth Muscle Cells

The pathologic events of vascular remodeling in the smooth muscle are predominantly mediated by HIF-1α (191). Ball et al. (192) using a model of chronic hypoxia, observed a decrease in wall thickness and muscularization in small pulmonary arteries of mice with specific deletion of HIF-1α in SMC. These effects attenuated the increased PVR and pulmonary hypertension. Accordingly, HIF-1α overexpression increased proliferation in human PASMC (193) suggesting that HIF-1α downstream effectors of vascular proliferation are involved in the development of PH.

The activation of the renin angiotensin system (RAS) has been associated with muscularization of the PASMC. Specifically, angiotensin converting enzyme (ACE) is responsible of transforming Angiotensin (Ang) I into Ang II, a hormone that stimulates proliferation and migration in the pulmonary vasculature (194). Conversely, ACE2 confers a compensatory mechanism by transforming Ang II in Ang1-7, a vasoprotective peptide with the opposite effects (i.e., antiproliferation, vasodilation) of Ang II. During hypoxia, ACE is directly upregulated, whereas ACE2 is indirectly downregulated by HIF-1α in PASMC (195). Therefore, the ratio between the expression and/or activity of both enzymes may show the severity of PAH. In support of this, patients with primary PH show increased activity of the RAS including ACE (196). Conversely, a negative correlation between the mPAP of patients with PAH and serum ACE2 has been described (197).

Gene expression of several components of the VEGF family including VEGF, PIGF, and its receptors (Flt-1 and Flk-1) are also dependent on HIF-1α activity. This family of growth factors is known for their proangiogenic and proliferative effects in multiple tissues including the lung (198, 199). Thus, the regulation of such factors in chronic hypoxic states determine the proliferative state and early evolution of PAH. For example, a functional polymorphism (rs833061T>C) that increases *VEGF* gene expression has been associated with an increased risk of developing PAH in the Chinese population (200). Accordingly, VEGF can exert its proliferative action in PASMC and LVEC contributing to the development and worsening of PAH (199, 201). The soluble receptor of the VEGF family, sFlt-1, and PIGF are also elevated in PAH patients and predicted survival (202, 203). Based on such findings, sFlt-1 and PIGF are proposed by Malhotra et al. as biomarkers elevated in the early phase of the disease.

Another important regulator of vascular tone, proliferation and survival dependent on HIF activity is endothelin 1 (ET-1). ET-1 can bind to ET_A_ receptors, mostly present in PASMC, and ET_B_ receptors which are predominantly expressed in endothelial cells. Some evidence has underpinned the relevance of ET-1 in the PASMC. For example, autocrine signaling of ET-1 appears to contribute to the muscularization and survival of human PASMCs (204). Moreover, a positive feedback loop between HIF-1α and ET-1 has been described in PASMCs. Chronic hypoxia increases ET-1 levels and upregulates HIF-1α synthesis while decreases degradation by PHD2 further favoring the HIF-1α-dependent expression of ET-1 (205). This suggests a maladaptation mechanism that perpetuates increased tone, proliferation, and survival of PASMC during chronic hypoxia. Perhaps this mechanism is a minor contributor in PAH pathology as the major production of ET-1 is located at the endothelial cells. The main effector of hypoxia in the pulmonary endothelium is HIF-2α (191) and partial deletion of HIF-2α in mice dampens ET-1 expression (190). Overall, ET-1 is deeply ingrained in PAH pathology and is finely regulated by both HIF subunits depending on the cell type. The importance of ET-1 in PAH is further revealed by clinical observations where PAH patients show increased ET-1 concentrations as the disease progresses (206–208). In fact, plasma levels of ET-1 are an independent predictor of the severity of PAH in the long-term (207, 209).

##### Pulmonary Artery Endothelial Cells

Even more relevant to the development and progression of PAH under hypoxia are the PAECs. The characteristic plexiform lesions in PAH that occlude the lumen of the pulmonary vessels are partly because of the deregulated proliferation of endothelial cells (210). HIF-2α is a key regulator of proliferation of PAECs in hypoxia as well as in normoxia when HIF-2α is artificially overexpressed (191). Endothelial-to-mesenchymal transition (EndoMT) is one of the mechanisms by which HIF-2α induce vascular remodeling and proliferation of PAEC in hypoxia-induced PAH (184). Moreover, in hif2a^+/−^ mice as well as in specific HIF-2α Knockout mice in PAECs the hypoxia-induced PAH is abolished (184, 190, 211). Accordingly, the deletion PHD2 in PAEC promotes vascular remodeling, enhances EndoMT and worsens the PAH (184, 212, 213). Thus, HIF-2α expression as well as its regulation by PHD2 are determinant factors for the development of PAH. This is consistent with genomic studies in humans living at high altitude where polymorphisms in both genes, *EPAS-1* for HIF-2α, and *EGLN1* for PHD2, are somewhat protecting from developing PAH in such hypoxic conditions (214–216).

Another important regulator of HIF-2α degradation is pVHL. In humans with Chuvash polycythemia, a single nucleotide polymorphism of pVHL (Arg200Trp) decreases the affinity for the hydroxylated HIF-α and impairs HIF degradation, thus, increasing HIF-2α accumulation and inducing the expression of other downstream effectors (217). Patients with Chuvash polycythemia show pulmonary vascular hyperresponsiveness to hypoxia and are at greater risk to develop PAH (218, 219). A murine model of Chuvash polycythemia spontaneously develops PAH mediated by HIF-2α and partial deletion of HIF-2α protected mice against the development of PAH (220). Thus, the lack of pVHL activity favors the development of PAH by increasing HIF-2α expression.

Finally, data is emerging describing a relationship of PAH with anemia (i.e., decreased red blood cell count and/or hemoglobin production), and iron deficiency. The hormone erythropoietin produced in the kidneys regulates red blood cell production. Iron homeostasis is dependent on several proteins for absorption, handling, and metabolism by the gastrointestinal system. A common link between both systems and PAH appears to be HIF-2α. On one hand, HIF-2α targets the *EPO* gene that encodes for erythropoietin under hypoxia (48, 221). Moreover, extrarenal HIF-2α is sufficient to stimulate erythropoietin production (221), suggesting that erythropoietin production might be stimulated by the overexpressed HIF-2α in hypertensive lungs. Accordingly, patients with PAH show high erythropoietin pulmonary levels that were associated with specific mitogenic effects in both PASMC and PAEC (222), independent of erythrocytosis (223). On the other hand, Iron regulation is intrinsically connected with oxygen transport and sensing as hemoglobin and PHDs, respectively, depend on Iron to function properly (described in section Oxygen Sensing). HIF-2α can regulate the key hormone of Iron homeostasis, hepcidin, produced in the liver, as well as be regulated by Iron regulatory proteins (IRPs), respectively (224, 225); a detailed review on this regulatory mechanism can be found in (226). Iron deficiency induced by the dysregulation of these factors ends in development of PAH (224, 227). Moreover, the Iron chelator agent deferrioxamine mimics hypoxia and rises the pulmonary arterial pressure in humans (228). Iron supplementation attenuated the normal pulmonary vasoconstrictive response to hypoxia and improved some functional measures in PAH patients (229, 230). Thus, regulation of Iron homeostasis and erythropoietin by HIF-2α can be collective determinants of PAH progression and severity as they indirectly reflect the activity of HIF-2α in other organs. Proof of this, is the inverse correlation of erythropoietin and Iron homeostasis in patients with PAH that was further associated with disease severity and poor clinical outcome (231, 232).

### Heart Failure

The primordial function of the heart is to ensure the constant supply of nutrients and oxygen to all tissues by continuously pumping blood to the system. Chronic impairments of cardiac contractility and load lead to heart failure (HF) and is often the pathologic result of multiple CV disorders (i.e., hypertension, coronary artery disease, valvulopathies, etc.; with the most common cause being cardiac damage from a myocardial infarction). HF has a complex pathophysiology that depends on the etiology and keeps evolving as more data emerges on the multiple molecular mechanisms involved in the process. Nevertheless, a somewhat simple classification of HF is according to the contractile performance of the heart and is used clinically; HF with reduced ejection Fraction (HFrEF) and HF with preserved ejection fraction (HFpEF), previously known as systolic and diastolic disfunction, respectively. In both cases, the chronic (mal)adaptive mechanisms of HF induce left ventricular (LV) remodeling further perpetuate the already impaired oxygen delivery to the heart and periphery (233, 234). Evidence on the regulation of the HIF pathway and downstream effectors by chronic hypoxia during HF is starting to emerge, making HIFs important players in the pathophysiology of HF. Here we focus on the growing evidence of the HIFs activity during the development of HF.

#### HIFs in Heart Failure

As previously described for other tissues, the regulation of HIFs in the heart is finely tuned by the duration of hypoxia. On one hand, chronic HIF-1α expression, measured using direct [overexpression (235, 236)] or indirect [PHD or VHL deletion (237, 238)] methods, shows HIF-1α induces cardiomyopathy in normoxic states and sudden cardiac decompensation when exposed to stressors such as pressure overload (236). On the other hand, specific deletion of HIF-1α in cardiomyocytes of mice show systolic and diastolic decompensation (239) and maladaptive hypertrophy with decreased angiogenesis when exposed to pressure overload (240). This seeming discrepancy, first, underlines the crucial role of HIF-1α regulation on the development of pathology and adaptation of the heart. Second, this evidence suggests that the activity of HIF-1α under pathologic conditions is limited to the early adaptive stage of chronic hypoxia as sustained expression beyond early adaptation, or complete absence of HIF-1α, is detrimental. Evidence in humans with HF is also consistent with such conclusions. Patients predominantly in the early stages of HF, based on the New York Heart Association (NYHA), functional class I/II, show increased expression of HIF-1α (236), whereas patients in end-stage HF (III/IV NYHA class), show decreased HIF-1α and PHD1 (241). Interestingly, HIF-3α and PHD3 were elevated in such patients suggesting that the orchestration of gene expression by chronic hypoxia is shifted to HIF-3α in the heart. Other mammals (i.e., sheep) exposed to years of chronic hypoxia have shown a similar reciprocal pattern in the heart where HIF-1α and HIF-3α are down- and upregulated, respectively (242). The regulation between both HIFs has been characterized in other tissues (243–246) and is consistent with a study in human cardiomyocytes where overexpression of HIF-3α inhibits HIF-1α expression and induces apoptosis (247). Thus, the expression of HIF-1α and HIF-3α is inversely proportional during HF.

#### Other Molecules downstream of HIF

##### B-Type Natriuretic Peptide

Currently one of the most widely used biomarkers for HF is B type natriuretic peptide (BNP). The production of BNP is predominantly stimulated by 3 main mechanisms: wall stretch, hormones (i.e., ET-1, Ang II) and inflammation (i.e., TNF-α, IL-6). The biologic activity of BNP is primarily mediated by the natriuretic peptide receptor -A (NPR-A) to induce diuresis, natriuresis, cell antiproliferation and antifibrotic effects, (248, 249). BNP activity has shown beneficial to the heart as an analog, nesiritide, improves the cardiac function of HF patients (250). Similarly, inhibition of the breakdown of BNP with a neprilysin inhibitor, sacubitril, attenuates cardiomyocyte cell death, hypertrophy, and impaired myocyte contractility (251). Emerging evidence has established hypoxia as another important stimulus for BNP secretion in cardiomyocytes (252–254). This secretion is dependent on HIF-1α binding to an identified HRE region of the human BNP promoter (254). Interestingly, the stretch-activated channels in cardiac myocytes, besides augmenting BNP secretion as part of the wall stretch mechanism (255), also increase HIF-1α even in normoxia (256), and both are blocked by gadolinium, a mechanosensitive channel inhibitor. Thus, the contribution of HIF on the release of BNP not only depends on the hypoxic mechanism induced by stretch but also by mechanical stretch directly, making BNP and HIF-1α highly relevant to HF pathology. Accordingly, BNP levels and the cleaved N-terminal of the prohormone BNP (NT-proBNP) are currently used as the biomarkers of choice to establish the chronicity and survival of HF patients as well as fatality prognosis in acute decompensated HF (257, 258). Another study positively correlated the BNP levels with HIF-1α in plasma further supporting the concept of hypoxia as a major mechanism of BNP release in patients with HFrEF (259). Thus, the chronic exposure to cardiac hypoxia and mechanical stress in HF favors BPN accumulation affecting the contractility of the heart and reflects the well-established predictive value of BNP as a biomarker.

##### Adrenomedullin

Originally discovered in pheochromocytoma tissue, adrenomedullin (AM) is expressed in multiple other cells including cardiomyocytes (260, 261) and coronary artery endothelial cells (262). AM is a potent vasodilator with antiapoptotic, diuretic and natriuretic properties (263). Similar to BNP, cardiac AM secretion increases by volume or pressure overload (264, 265). Hypoxia is also another important factor for AM release in the heart (266). Accordingly, the promoter region of the *ADM* gene in cardiomyocytes contains the HIF-1 consensus sites that respond to hypoxia (260). Furthermore, hypoxia increases the protein expression of both endothelial PAS domain-containing protein 1 (EPAS1), also known as HIF-2α, and AM in cardiomyocytes in a similar time-dependent fashion. When EPAS1 is overexpressed in normoxic conditions, AM levels increase proportionately (261). Therefore, this suggests the *ADM* gene is a target of HIFs. A more intricate interaction has been shown where AM upregulation by a HIF-1α-independent hypoxic mechanism enhances HIF-1α stabilization and activity by inhibiting PHDs and inducing translocation of HIF-1α to the nucleus in multiple human cell types (267, 268). Taken together, this data suggests that AM and HIF-1 work together under a positive feedback loop in hypoxic conditions. The relevance of this mechanism in heart failure has not been explored. The overlapping profile of AM with HIFs in HF is detrimental as these patients show elevated AM that, (similar to BNP and HIF-1α expression), positively correlate with the NYHA classification (i.e., severity of the disease is proportional to the AM levels) (269–271). Moreover, AM has been shown as an independent predictor of prognosis in HF (272) suggesting that AM levels in HF correlate well with the development and progression of the disease.

## Conclusions

The unique interaction of oxygen with eukaryotic cells has made possible the formation of highly complex living organisms by providing the means to increase energy production and metabolism. The findings of Semenza, Ratcliffe, Kaelin, and others about HIFs and its regulators, PHDs and VHL, during hypoxia have paved the way to understand in greater depth the mechanisms involved in fundamental cellular processes. The versatility of these proteins to respond to hypoxia and activate a set of genes in a particular tissue show a fine cellular regulation for adaptation in acute vs. chronic settings. As our understanding on the PHD/HIF-targeted gene-effect axis keeps expanding we will be able to identify new pathologic processes that accurately reflect the affected tissue in a CV disease and provide targets for a better therapeutic approach. This is already the case in a related illness, chronic kidney disease, where one of the early complications is anemia due to lack of EPO production by kidney interstitial fibroblasts-like cells. Using a PHD inhibitor, Roxadustat, in these patients allow HIFs to accumulate and, thus, enhance EPO secretion and normalize Iron homeostasis, breaking the vicious cycle of anemia that affects other CV organs and increase the morbidity and mortality rate in such patients (273, 274). As detailed in this review, hypoxia is one of the main events that trigger multiple CV diseases. The downstream mechanisms activated by hypoxia in the CV system vary according to the affected organ/cell type and the timespan of the ischemic insult. This in turn triggers the expression of a particular set of genes from over a thousand HIF-dependent genes. Identification of a unique set of HIF-dependent genes affected by hypoxia in CV diseases could define the patterns of gene expression per tissue [as illustrated by Semenza (275)]. Accurate identification of such genes might reveal the developmental stage of the disease and the gene “fingerprint” of the most affected cell type(s). This could further allow us to identify the injured tissue at earlier stages of the disease to provide for early intervention and determination of the best therapeutic choice for patients with CV diseases.

## Author Contributions

EYLGR was primarily responsible for writing the first draft of most sections of the review. CV and RB were responsible for editing the manuscript. All authors contributed equally to the concept and outline of the review.

## Conflict of Interest

The authors declare that the research was conducted in the absence of any commercial or financial relationships that could be construed as a potential conflict of interest.

## Publisher's Note

All claims expressed in this article are solely those of the authors and do not necessarily represent those of their affiliated organizations, or those of the publisher, the editors and the reviewers. Any product that may be evaluated in this article, or claim that may be made by its manufacturer, is not guaranteed or endorsed by the publisher.
